# Pediatric Traumatic Injuries Due to Agrarian Hay-Hole Falls

**DOI:** 10.7759/cureus.51892

**Published:** 2024-01-08

**Authors:** Ae Lim Yang, Oliver D Mrowczynski, Ryan J Jafrani, Junjia Zhu, Mark Dias, Elias Rizk

**Affiliations:** 1 Medicine, Penn State Health Milton S. Hershey Medical Center, Hershey, USA; 2 Neurosurgery, Penn State Health Milton S. Hershey Medical Center, Hershey, USA; 3 Statistics, Penn State Health Milton S. Hershey Medical Center, Hershey, USA; 4 Neurological Surgery, Penn State Health Milton S. Hershey Medical Center, Hershey, USA

**Keywords:** hay-hole fall, length of stay, skull fracture, hay-holes, child trauma

## Abstract

Hay-holes are a design feature in many traditionally built barns that serve as a portal through which stored hay is passed to the lower level where animals are fed. Unfortunately, children sometimes fall through the hay-hole to the concrete or packed earth below. Available data on the frequency and types of hay-hole injuries is limited. The purpose of this study was to better characterize the resultant injuries and identify prognostic factors that predict outcomes from them. We performed a retrospective review of 53 children admitted to the Penn State Hershey Children's Hospital at the Penn State Hershey Medical Center with injuries due to a fall through a hay-hole over 15 years. Compared to urban trauma, hay-hole falls more frequently involve younger children and craniofacial injuries. Although they may result in significant injuries, they are rarely fatal. Greater fall height is associated with longer length of stay (LOS) but not with a greater frequency of intubation, intracranial hemorrhage, or skull fracture. A re-examination of barn design may help to reduce the frequency of this injury type.

## Introduction

Unintentional falls were the leading cause of nonfatal injury among children 0-4 years from 2000 to 2015 in the United States [1]. Prior guidelines and prevention efforts have focused on urban-based populations, with common mechanisms being falls from roofs, windows, and balconies [2,3]. However, agricultural occupations rank among the most hazardous professions in the United States, with significant associated mortality and morbidity annually [4,5]. Children living on farms are at risk of multiple unique injury types. An unexpected common source of injury, particularly in Amish and Mennonite communities, is falls involving hay-holes, as noted in multiple sources [5].

Hay-holes are a standard design feature in many traditionally built barns, particularly those in the upper midwestern United States and Canada, that serve as a portal through which stored hay is passed to the lower level where animals are fed [5]. Unfortunately, children sometimes fall through the hay-hole to the concrete or packed earth below [5]. Available data on the frequency and types of hay-hole injuries is limited. The purpose of this study is to better characterize the resultant injuries and identify prognostic factors that predict outcomes from them.

## Materials and methods

Study sample

We performed a retrospective review of 53 children admitted to the Penn State Hershey Children's Hospital at the Penn State Hershey Medical Center with injuries due to a fall through a hay-hole over 15 years. Electronic health records were reviewed for patient demographics, the height of fall, injuries, interventions, complications, and hospital course. The primary outcome variable in this study was the patients' length of stay (LOS) in days. We analyzed the height of fall and resultant complications. 

Statistical methods

Descriptive statistics for quantitative variables (mean, standard deviation) and categorical variables (frequencies, proportions) were used to summarize patients' demographics and baseline characteristics at admission. The linear associations between quantitative exploratory variables and the primary quantitative outcome variable were illustrated using scatter plots and tested using Pearson correlation coefficients. The associations between other binary categorical exploratory variables and the outcome were explored using a two-sample t-test. The associations between the dichotomized LOS and its possible predictors were studied using Fisher's exact test or two-sample t-test when appropriate. Due to this study's limited sample size and exploratory nature, multi-variable regression methods were not used to examine the bivariate association results. All analyses were performed using the statistical programming language R version 4.0.2 (R Foundation, Vienna, Austria) [6]. All tests were two-sided, and the statistical significance level was set at 0.05.

## Results

Patient demographics and admission characteristics

Patient demographics and admission characteristics for the 53 patients are listed in Table 1.

**Table 1:** Patient and admission characteristics GCS: Glasgow Coma Scale; ICH: intracranial hemorrhage

Variable	N (%)
Female	11 (20.8%)
Male	42 (79.2%)
Age, mean (range), years	4.8 (1.4-12.3)
Amish/Mennonite	53 (100%)
Height of fall, mean (range), feet	9.5 (6-19)
Intubated on arrival	13 (24.5%)
GCS on arrival, mean (range)	12.1 (3-15)
GCS 14 or less	23 (43.4%)
GCS of 15	30 (56.6%)
GCS on discharge, mean (range)	14.5 (3-15)
Loss of consciousness	
Positive	23 (43.4%)
Unknown	30 (56.6%)
Skull fracture	38 (71.7%)
ICH	21 (39.6%)
Epidural hematoma	3 (5.7%)
Subdural hematoma	14 (26.4%)
Intraparenchymal hemorrhage	4 (7.5%)
Craniofacial injury (ICH or skull fracture or facial injury)	45 (84.9%)
Spine injury fracture of ligamentous injury	3 (5.7%)
Length of stay, mean (range), days	2.1 (0-8)
Less than or equal to a day stay	24 (45.3%)
More than a day stay	29 (54.7%)
Mortality	1 (1.9%)

All children were Amish or Mennonite and predominantly male (79.2%), with a mean age of 4.8 years (range 1.4-12.3). The average fall height was 9.5 feet (range 6-19 feet). Twenty-three patients (43.4%) had a loss of consciousness at the time of fall. Thirteen patients (24.5%) were intubated at the time of arrival. The average Glasgow Coma Scale (GCS) on arrival was 12.1, with 23 patients (43.4%) having GCS of 14 or less. 

Injuries and outcomes

Patient injuries and outcomes are listed in Table 2. Thirty-eight patients (71.7%) had skull fractures, and three patients (5.7%) had a spinal fracture or ligamentous injury. Twenty-one patients (39.6%) had an intracranial hemorrhage, the most common type being a subdural hematoma. No patients required surgical intervention. The average hospital LOS was 2.1 days, with 24 (45.3%) patients able to be discharged home the next day. Fifty-one patients were discharged to home, and one was discharged to a rehabilitation facility; there was one patient death, attributed to hypoxic-ischemic brain injury from unrecognized esophageal intubation during transport.

**Table 2:** Height of fall vs. length of stay and other complications SD: standard deviation; GCS: Glasgow Coma Scale

Variable	Height of fall (feet)		p-value
	Mean (SD)	Median (range)	
Overall	9.5 (2.5)	9 (6-19)	
Length of stay			0.0479
Less than or equal to a day stay	8.8 (1.3)	9 (7-12)	
More than a day stay	10.1 (3.1)	9 (6-19)	
Gender			0.8339
Male	9.6 (2.2)	9 (6-15)	
Female	9.4 (3.3)	8 (7-19)	
Loss of consciousness			0.2501
Yes	10.1 (2.6)	9.5 (7-15)	
No	9.2 (2.4)	9 (6-19)	
Intubation on arrival			0.5458
Yes	10 (2.9)	9 (6-15)	
No	9.4 (2.4)	9 (7-19)	
GCS on arrival			0.0553
GCS of 14 or less	10.4 (3.3)	10 (6-19)	
GCS of 15	8.9 (1.4)	9 (7-14)	
Skull fracture			0.4533
Yes	9.3 (2.1)	9 (7-15)	
No	10.1 (3.4)	9.5 (6-19)	
Intracranial hemorrhage			0.486
Yes	9.9 (2.8)	8.5 (6-15)	
No	9.3 (2.3)	9 (7-19)	

Factors associated with hospital LOS and other complications

As shown in Table 3, increased hospital LOS was associated with a presenting GCS of 14 or lower (p=0.009), intubation status on arrival (p=0.008), intracranial bleed (p=0.0329), and increased fall height (p=0.048). There was a trend toward an association between fall height and presenting GCS (p=0.06) but was not independently associated with intubation status upon arrival, presence of intracranial hemorrhage, or skull fracture. The presence of a skull fracture was not associated with the length of LOS.

**Table 3:** Factors affecting the length of stay SD: standard deviation; GCS: Glasgow Coma Scale; ICH: intracranial hemorrhage

Variable	Categories	Overall	Less than or equal to a day stay	More than a day stay	p-value
Age when admitted	Mean (SD)	4.8 (2.5)	4.6 (2.7)	5 (2.2)	0.5712
	Median (range)	5.2 (1.4-12.3)	4.2 (1.4-11.7)	5.3 (2.2-12.3)	
Gender	Female	11 (21.2%)	5 (45.5%)	6 (54.5%)	1
	Male	41 (78.8%)	19 (46.3%)	22 (53.7%)	
Loss of consciousness	Yes	22 (42.3%)	7 (31.8%)	15 (68.2%)	0.1351
	No	30 (56.6%)	17 (56.7%)	13 (43.3%)	
Intubation on arrival	Yes	12 (23.1%)	1 (8.3%)	11 (91.7%)	0.0077
	No	40 (76.9%)	23 (57.5%)	17 (42.5%)	
GCS on arrival	GCS of 14 or less	22 (42.3%)	5 (22.7%)	17 (77.3%)	0.0009
	GCS of 15	30 (57.7%)	19 (63.3%)	11 (36.7%)	
Spine injury fracture or ligamentous injury	Yes	3 (5.8%)	2 (66.7%)	1 (33.3%)	0.8905
	No	49 (94.2%)	22 (44.9%)	27 (55.1%)	
Intracranial hemorrhage	Yes	20 (38.5%)	5 (25%)	15 (75%)	0.0329
	No	32 (61.5%)	19 (59.4%)	13 (40.6%)	
ICH type	Epidural hematoma	3 (5.8%)	0 (0%)	3 (100%)	0.0799
	Intraparenchymal hemorrhage	3 (5.8%)	1 (33.3%)	2 (66.7%)	
	Subdural hematoma	14 (26.9%)	4 (28.6%)	10 (71.4%)	
Skull fracture	Yes	37 (71.2%)	16 (43.2%)	21 (56.8%)	0.7232
	No	15 (28.8%)	8 (53.3%)	7 (46.7%)	

## Discussion

There are estimated to be approximately 1 million children and adolescents residing on 2.2 million farms across the United States [7], but only one-third of farm-related injuries are work-related [8]. Children who are members of the Anabaptist and Pietist sects, commonly referred to as the Amish and Mennonites, are at a higher risk of farm-related injuries [9,10]. Hay-hole falls are unique in that they occur secondary to a particular barn design. It is likely that many of these children were injured while playing rather than working on the farm, since the mean age of the patients in the study was 4.8 years. The majority (79.2%) of the victims in our study were boys, consistent with the previous literature [10].

Also consistent with prior studies [7,11], craniofacial injuries predominated in our series and included intracranial hemorrhages, skull fractures, and facial fractures. In comparison, falls of 15 feet or less among urban populations report an older age distribution, lower frequency of craniofacial injuries, and higher frequency of truncal injuries and/or extremity fractures [12-15]. The younger age of children suffering hay-hole injuries, whose heads represent a greater proportion of body mass and whose developmental age may make them less able to protect themselves from injury; the hard surface of the barn floor; and the mechanics of hay-hole injuries in which a child falls through an opening in the floor rather than through a vertically oriented window could influence the pattern of injury [11,14-16]. Finally, while reports of fall height are often unreliable, the median reported height of hay-hole fall is consistent with the specifications of barn design.

The average height of fall was 9.5 feet which is consistent with the specifications of Amish and Mennonite barn design. Increased fall height was associated with greater LOS (p=0.048) as well as a trend toward lower presenting GCS (p=0.055), but not with intubation status upon arrival, presence of intracranial hemorrhage, or skull fracture (p all >0.05). It is therefore unclear whether greater fall height is absolutely associated with more severe injuries. Our study also demonstrated that increased LOS was also associated with a presenting GCS of ≤14 (p=0.009), intubation status on arrival (p=0.008), and an intracranial hemorrhage (p=0.033). It is therefore unclear whether greater fall height is absolutely associated with more severe injuries.

This study has several limitations including its retrospective design, single institution and unusual population demographic, and small sample size. Some information came from the observers at the scene, including other children. We could not include patients who may have sustained hay-hole falls but were treated at outside hospitals but not transferred to our institution or who sustained minor injuries that did not require evaluation. However, given the widespread use of this particular barn design (the Pennsylvania forebay bank barn) and the strict adherence of many agrarian communities to tradition, it is likely that our experience applies to other geographic areas.

## Conclusions

Pediatric hay-hole falls represent a specific injury mechanism. They more frequently involve younger children and craniofacial injuries compared with urban falls. Although they may result in significant injuries, they are rarely fatal. Greater fall height is associated with longer LOS but not with a greater frequency of intubation, intracranial hemorrhage, or skull fracture. A re-examination of barn design may help to reduce the frequency of this injury type.
